# Dichlorido(10,11,12,13-tetra­hydro-4,5,9,14-tetra­azabenzo[*b*]triphenyl­ene)cadmium(II) hemihydrate

**DOI:** 10.1107/S1600536808041305

**Published:** 2008-12-13

**Authors:** Chun-Xiang Li, Xiu-Ying Li, Chun-Bo Liu, Yong-Sheng Yan, Guang-Bo Che

**Affiliations:** a School of Chemistry and Chemical Engineering, Jiangsu University, Zhenjiang 212013, People’s Republic of China; b Department of Chemistry, Jilin Normal University, Siping 136000, People’s Republic of China

## Abstract

In the title compound, [CdCl_2_(C_18_H_14_N_4_)_2_]·0.5H_2_O, the Cd atom assumes a distorted octa­hedral *trans*-CdCl_2_N_4_ geometry arising from its coordination by two *N*,*N*′-bidentate 10,11,12,13-tetra­hydro-4,5,9,14-tetra­azabenzo[*b*]triphenyl­ene (TBBT) mol­ecules and two chloride ions. In the crystal, π–π aromatic stacking inter­actions between adjacent TTBT rings are seen, with a centroid–centroid distance of 3.604 (3) Å. An O—H⋯Cl hydrogen bond between the half-occupied water molecule and one chloride ion also occurs.

## Related literature

For the synthesis of the ligand, see: Che *et al.* (2006 ▶). For related structures and background, see: Wei *et al.* (2007 ▶); Che *et al.* (2008 ▶); Xu *et al.* (2008 ▶).
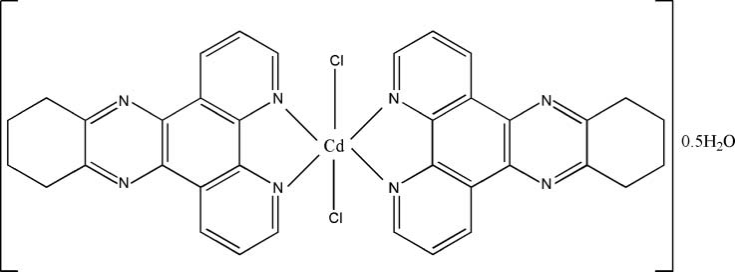


## Experimental

### 

#### Crystal data

[CdCl_2_(C_18_H_14_N_4_)_2_]·0.5H_2_O
*M* *_r_* = 764.97
Monoclinic, 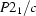

*a* = 15.369 (4) Å
*b* = 14.237 (3) Å
*c* = 16.506 (4) Å
β = 116.561 (3)°
*V* = 3230.4 (13) Å^3^
*Z* = 4
Mo *K*α radiation
μ = 0.88 mm^−1^
*T* = 292 (2) K
0.29 × 0.20 × 0.09 mm

#### Data collection

Bruker SMART APEX CCD diffractometer
Absorption correction: multi-scan (*SADABS*; Bruker, 1998 ▶) *T*_min_ = 0.811, *T*_max_ = 0.924
17793 measured reflections
6343 independent reflections
4692 reflections with *I* > 2σ(*I*)
*R*_int_ = 0.045

#### Refinement

*R*[*F* ^2^ > 2σ(*F* ^2^)] = 0.046
*wR*(*F* ^2^) = 0.106
*S* = 1.01
6343 reflections
439 parameters
3 restraints
H atoms treated by a mixture of independent and constrained refinement
Δρ_max_ = 0.64 e Å^−3^
Δρ_min_ = −0.36 e Å^−3^

### 

Data collection: *SMART* (Bruker, 1998 ▶); cell refinement: *SAINT* (Bruker, 1998 ▶); data reduction: *SAINT*; program(s) used to solve structure: *SHELXS97* (Sheldrick, 2008 ▶); program(s) used to refine structure: *SHELXL97* (Sheldrick, 2008 ▶); molecular graphics: *SHELXTL-Plus* (Sheldrick, 2008 ▶); software used to prepare material for publication: *SHELXL97*.

## Supplementary Material

Crystal structure: contains datablocks global, I. DOI: 10.1107/S1600536808041305/hb2870sup1.cif

Structure factors: contains datablocks I. DOI: 10.1107/S1600536808041305/hb2870Isup2.hkl

Additional supplementary materials:  crystallographic information; 3D view; checkCIF report

## Figures and Tables

**Table:** 

Cd—N1	2.390 (3)
Cd—N2	2.445 (3)
Cd—N5	2.367 (3)
Cd—N6	2.532 (3)
Cd—Cl1	2.4886 (12)
Cd—Cl2	2.5067 (11)

**Table:** 

N5—Cd—N1	146.20 (10)
N5—Cd—N2	86.77 (10)
N5—Cd—Cl1	101.33 (8)
N1—Cd—Cl1	96.70 (8)
N1—Cd—Cl2	107.93 (8)
Cl1—Cd—Cl2	104.21 (4)

**Table 2:** Hydrogen-bond geometry (Å, °)

*D*—H⋯*A*	*D*—H	H⋯*A*	*D*⋯*A*	*D*—H⋯*A*
O1*W*—H1*WB*⋯Cl2^i^	0.81 (2)	2.79 (7)	3.326 (7)	126 (7)

Symmetry code: (i) 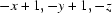.

**Fig. 1.**
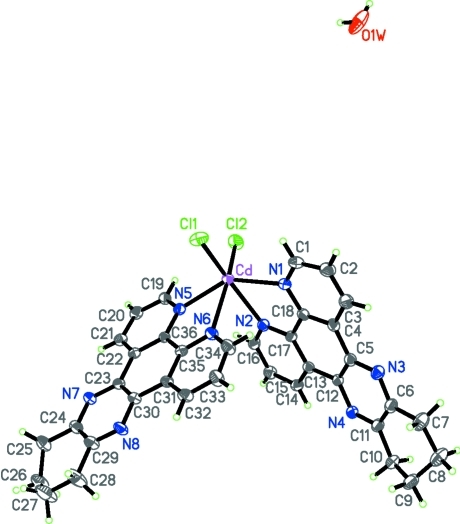
The molecular structure of (I). Displacement ellipsoids are drawn at the 30% probability level (arbitrary spheres for the H atoms).

**Fig. 2.**
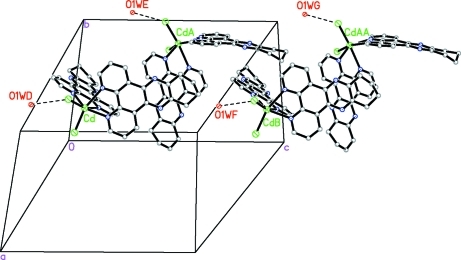
A view of the crystal packing, showing the π-π stacking and intermolecular hydrogen bonds interaction. H atoms have been omitted. [Symmetry codes: (A) x, -y + 5/2, z - 1/2; (B) x, y, z + 1; (C) x, -y + 5/2, z - 3/2; (AA) x, -y + 5/2, z - 3/2; (D) -x + 1, -y + 1, -z; (E) -x + 1, y + 3/2, -z + 1/2; (F) -x + 1, -y + 1, -z + 1; (G) -x + 1, y + 3/2, -z + 3/2]

## supplementary crystallographic information

### Comment

The rational design and construction of new coordination supramolecular compounds based on assembly of metal ions and multifunctional organic ligands are of great current interest (Wei *et al.*, 2007). 1,10-Phenanthroline and its derivatives, as a series of important ligands with numerous uses, have been extensively studied in the chemistry of coordination polymers (Che *et al.*, 2008; Xu *et al.*, 2008). Hereby, we have prepared the title compound, (I), (Fig. 1) or [Cd(TTBT)_2_Cl_2_].0.5H_2_O (I), where TTBT = 10,11,12,13-tetrahydro-4,5,9,14-tetraazabenzo[*b*]triphenylene.

The Cd^II^ atom is coordinated by four N atoms from two bidentate TTBT molecules, and two Cl^-^ anions, resulting in a distorted octahedral coordination geometry (Table 1). Neighbouring mononuclear units contact through π-π interaction between two TTBT ligands (centroid separation = 3.604 (3) Å) and intermolecular hydrogen bonds (Table 2), leading to a network structure (Fig. 2).

### Experimental

The TTBT ligand was synthesized according to the literature method of Che *et al.* (2006). Compound (I) was hydrothermally synthesized under autogenous pressure: a mixture of TTBT, CdCl_2_ and water in a molar ratio of 2:1:5000 was sealed in a Teflon-lined autoclave and heated to 423 K for 3 d. Upon cooling and opening the bomb, yellow blocks of (I) were obtained (79% yield based on Cd).

### Refinement

The water molecule is disordered with a site-occupancy factor of 0.5. A l l H atoms on C atoms were positioned geometrically (C—H = 0.93 Å) and refined as riding with *U*_iso_(H)= 1.2*U*_eq_(C).

### Crystal data

**Table:**

[CdCl_2_(C_18_H_14_N_4_)_2_]·0.5H_2_O	*F*(000) = 1548
*M**_r_* = 764.97	*D*_x_ = 1.573 Mg m^−^^3^
Monoclinic, *P*2_1_/*c*	Mo *K*α radiation, λ = 0.71073 Å
Hall symbol: -P 2ybc	Cell parameters from 3827 reflections
*a* = 15.369 (4) Å	θ = 1.5–26.1°
*b* = 14.237 (3) Å	µ = 0.88 mm^−^^1^
*c* = 16.506 (4) Å	*T* = 292 K
β = 116.561 (3)°	Block, yellow
*V* = 3230.4 (13) Å^3^	0.29 × 0.20 × 0.09 mm
*Z* = 4	

### Data collection

**Table:**

Bruker SMART APEX CCD diffractometer	6343 independent reflections
Radiation source: fine-focus sealed tube	4692 reflections with *I* > 2σ(*I*)
graphite	*R*_int_ = 0.045
ω scan	θ_max_ = 26.1°, θ_min_ = 2.0°
Absorption correction: multi-scan (*SADABS*; Bruker, 1998)	*h* = −19→16
*T*_min_ = 0.811, *T*_max_ = 0.924	*k* = −17→17
17793 measured reflections	*l* = −17→20

### Refinement

**Table:**

Refinement on *F*^2^	Primary atom site location: structure-invariant direct methods
Least-squares matrix: full	Secondary atom site location: difference Fourier map
*R*[*F*^2^ > 2σ(*F*^2^)] = 0.046	Hydrogen site location: inferred from neighbouring sites
*wR*(*F*^2^) = 0.106	H atoms treated by a mixture of independent and constrained refinement
*S* = 1.01	*w* = 1/[σ^2^(*F*_o_^2^) + (0.0498*P*)^2^] where *P* = (*F*_o_^2^ + 2*F*_c_^2^)/3
6343 reflections	(Δ/σ)_max_ = 0.002
439 parameters	Δρ_max_ = 0.64 e Å^−^^3^
3 restraints	Δρ_min_ = −0.35 e Å^−^^3^

### Special details

**Table:**

Geometry. All e.s.d.'s (except the e.s.d. in the dihedral angle between two l.s. planes) are estimated using the full covariance matrix. The cell e.s.d.'s are taken into account individually in the estimation of e.s.d.'s in distances, angles and torsion angles; correlations between e.s.d.'s in cell parameters are only used when they are defined by crystal symmetry. An approximate (isotropic) treatment of cell e.s.d.'s is used for estimating e.s.d.'s involving l.s. planes.
Refinement. Refinement of *F*^2^ against ALL reflections. The weighted *R*-factor *wR* and goodness of fit *S* are based on *F*^2^, conventional *R*-factors *R* are based on *F*, with *F* set to zero for negative *F*^2^. The threshold expression of *F*^2^ > σ(*F*^2^) is used only for calculating *R*-factors(gt) *etc*. and is not relevant to the choice of reflections for refinement. *R*-factors based on *F*^2^ are statistically about twice as large as those based on *F*, and *R*- factors based on ALL data will be even larger.

### Fractional atomic coordinates and isotropic or equivalent isotropic displacement parameters (Å^2^)

**Table:**

	*x*	*y*	*z*	*U*_iso_*/*U*_eq_	Occ. (<1)
C1	0.7654 (3)	0.7883 (3)	0.4325 (3)	0.0486 (11)	
H1	0.7889	0.7534	0.3989	0.058*	
C2	0.7844 (3)	0.7566 (3)	0.5187 (3)	0.0533 (12)	
H2	0.8175	0.7004	0.5407	0.064*	
C3	0.7538 (3)	0.8086 (3)	0.5702 (3)	0.0484 (11)	
H3	0.7672	0.7893	0.6284	0.058*	
C4	0.7019 (3)	0.8921 (3)	0.5343 (3)	0.0369 (9)	
C5	0.6722 (3)	0.9545 (3)	0.5867 (3)	0.0359 (9)	
C6	0.6797 (3)	0.9972 (3)	0.7240 (3)	0.0433 (10)	
C7	0.7191 (4)	0.9787 (4)	0.8231 (3)	0.0611 (13)	
H7A	0.7892	0.9859	0.8509	0.073*	
H7B	0.7052	0.9140	0.8317	0.073*	
C8	0.6783 (4)	1.0427 (4)	0.8718 (3)	0.0681 (15)	
H8A	0.6151	1.0196	0.8624	0.082*	
H8B	0.7211	1.0415	0.9363	0.082*	
C9	0.6683 (4)	1.1413 (3)	0.8380 (3)	0.0619 (13)	
H9A	0.7316	1.1647	0.8478	0.074*	
H9B	0.6447	1.1809	0.8718	0.074*	
C10	0.5988 (3)	1.1464 (3)	0.7385 (3)	0.0512 (11)	
H10A	0.5333	1.1344	0.7305	0.061*	
H10B	0.6002	1.2094	0.7166	0.061*	
C11	0.6228 (3)	1.0775 (3)	0.6829 (3)	0.0402 (10)	
C12	0.6174 (3)	1.0346 (3)	0.5469 (3)	0.0334 (9)	
C13	0.5908 (3)	1.0569 (3)	0.4530 (3)	0.0336 (9)	
C14	0.5329 (3)	1.1345 (3)	0.4085 (3)	0.0374 (9)	
H14	0.5085	1.1734	0.4388	0.045*	
C15	0.5125 (3)	1.1527 (3)	0.3205 (3)	0.0417 (10)	
H15	0.4729	1.2030	0.2895	0.050*	
C16	0.5526 (3)	1.0941 (3)	0.2781 (3)	0.0412 (10)	
H16	0.5397	1.1074	0.2186	0.049*	
C17	0.6251 (3)	1.0006 (2)	0.4041 (2)	0.0314 (8)	
C18	0.6816 (3)	0.9167 (2)	0.4450 (3)	0.0340 (9)	
C19	0.6633 (3)	1.0949 (3)	0.1052 (3)	0.0381 (9)	
H19	0.6189	1.0485	0.0724	0.046*	
C20	0.6692 (3)	1.1744 (3)	0.0594 (3)	0.0423 (10)	
H20	0.6289	1.1812	−0.0023	0.051*	
C21	0.7352 (3)	1.2427 (3)	0.1064 (2)	0.0393 (9)	
H21	0.7416	1.2957	0.0765	0.047*	
C22	0.7929 (3)	1.2323 (3)	0.1993 (2)	0.0324 (8)	
C23	0.8625 (3)	1.3037 (3)	0.2537 (3)	0.0347 (9)	
C24	0.9349 (3)	1.4456 (3)	0.2630 (3)	0.0431 (10)	
C25	0.9495 (4)	1.5326 (3)	0.2194 (3)	0.0556 (12)	
H25A	0.9969	1.5195	0.1971	0.067*	
H25B	0.8886	1.5485	0.1678	0.067*	
C26	0.9830 (4)	1.6144 (3)	0.2810 (4)	0.0764 (16)	
H26A	0.9284	1.6397	0.2886	0.092*	
H26B	1.0055	1.6629	0.2536	0.092*	
C27	1.0627 (5)	1.5906 (4)	0.3716 (4)	0.099 (2)	
H27A	1.1225	1.5878	0.3655	0.119*	
H27B	1.0691	1.6428	0.4117	0.119*	
C28	1.0573 (4)	1.5080 (4)	0.4159 (4)	0.092 (2)	
H28A	1.0383	1.5252	0.4626	0.110*	
H28B	1.1221	1.4812	0.4460	0.110*	
C29	0.9892 (3)	1.4328 (3)	0.3584 (3)	0.0471 (11)	
C30	0.9170 (3)	1.2911 (3)	0.3473 (3)	0.0360 (9)	
C31	0.9058 (3)	1.2065 (3)	0.3907 (2)	0.0336 (9)	
C32	0.9576 (3)	1.1909 (3)	0.4843 (3)	0.0435 (10)	
H32	1.0017	1.2354	0.5213	0.052*	
C33	0.9430 (3)	1.1105 (3)	0.5203 (3)	0.0508 (12)	
H33	0.9760	1.0997	0.5824	0.061*	
C34	0.8784 (3)	1.0450 (3)	0.4638 (3)	0.0480 (11)	
H34	0.8704	0.9896	0.4896	0.058*	
C35	0.8396 (3)	1.1366 (3)	0.3384 (2)	0.0326 (9)	
C36	0.7818 (2)	1.1504 (2)	0.2411 (2)	0.0296 (8)	
N1	0.7157 (2)	0.8656 (2)	0.3962 (2)	0.0389 (8)	
N2	0.6075 (2)	1.0210 (2)	0.3179 (2)	0.0357 (7)	
N3	0.7024 (2)	0.9358 (2)	0.6756 (2)	0.0424 (8)	
N4	0.5910 (2)	1.0950 (2)	0.5956 (2)	0.0382 (8)	
N5	0.7177 (2)	1.0813 (2)	0.1935 (2)	0.0350 (7)	
N6	0.8272 (2)	1.0554 (2)	0.3753 (2)	0.0376 (8)	
N7	0.8720 (2)	1.3817 (2)	0.2118 (2)	0.0393 (8)	
N8	0.9807 (2)	1.3570 (2)	0.3997 (2)	0.0443 (9)	
O1W	0.4540 (7)	0.1941 (8)	0.0710 (4)	0.104 (3)	0.50
Cd	0.71099 (2)	0.938412 (19)	0.263814 (19)	0.03564 (11)	
Cl1	0.85128 (8)	0.85170 (8)	0.26317 (8)	0.0598 (3)	
Cl2	0.56616 (8)	0.87531 (8)	0.12900 (7)	0.0582 (3)	
H1WA	0.432 (8)	0.137 (3)	0.057 (6)	0.070*	0.50
H1WB	0.453 (7)	0.218 (5)	0.026 (4)	0.070*	0.50

### Atomic displacement parameters (Å^2^)

**Table:**

	*U*^11^	*U*^22^	*U*^33^	*U*^12^	*U*^13^	*U*^23^
C1	0.059 (3)	0.034 (2)	0.056 (3)	0.003 (2)	0.029 (2)	−0.005 (2)
C2	0.066 (3)	0.037 (2)	0.055 (3)	0.012 (2)	0.026 (3)	0.007 (2)
C3	0.058 (3)	0.042 (2)	0.044 (3)	0.005 (2)	0.022 (2)	0.006 (2)
C4	0.040 (2)	0.031 (2)	0.038 (2)	0.0003 (17)	0.0160 (19)	0.0055 (18)
C5	0.036 (2)	0.037 (2)	0.033 (2)	−0.0046 (17)	0.0145 (18)	−0.0004 (17)
C6	0.045 (3)	0.051 (3)	0.036 (2)	−0.004 (2)	0.020 (2)	−0.002 (2)
C7	0.059 (3)	0.083 (3)	0.038 (3)	0.008 (3)	0.018 (2)	0.003 (2)
C8	0.071 (4)	0.095 (4)	0.039 (3)	0.016 (3)	0.025 (3)	−0.001 (3)
C9	0.071 (3)	0.074 (3)	0.049 (3)	−0.005 (3)	0.035 (3)	−0.015 (3)
C10	0.063 (3)	0.055 (3)	0.045 (3)	−0.002 (2)	0.034 (2)	−0.007 (2)
C11	0.042 (2)	0.045 (2)	0.036 (2)	−0.0040 (19)	0.020 (2)	−0.0037 (19)
C12	0.036 (2)	0.033 (2)	0.033 (2)	−0.0056 (16)	0.0169 (19)	−0.0023 (16)
C13	0.032 (2)	0.034 (2)	0.033 (2)	−0.0075 (17)	0.0129 (17)	−0.0027 (17)
C14	0.038 (2)	0.035 (2)	0.041 (2)	0.0020 (17)	0.0183 (19)	−0.0033 (18)
C15	0.041 (2)	0.040 (2)	0.040 (2)	0.0067 (18)	0.015 (2)	0.0067 (19)
C16	0.043 (2)	0.043 (2)	0.035 (2)	0.000 (2)	0.015 (2)	0.0060 (19)
C17	0.031 (2)	0.032 (2)	0.031 (2)	−0.0031 (16)	0.0133 (17)	−0.0038 (17)
C18	0.034 (2)	0.029 (2)	0.040 (2)	−0.0020 (16)	0.0178 (19)	−0.0009 (17)
C19	0.037 (2)	0.044 (2)	0.030 (2)	−0.0112 (18)	0.0113 (18)	−0.0072 (18)
C20	0.040 (2)	0.053 (3)	0.029 (2)	−0.001 (2)	0.0109 (19)	−0.003 (2)
C21	0.043 (2)	0.041 (2)	0.031 (2)	0.0065 (19)	0.0146 (19)	0.0010 (18)
C22	0.033 (2)	0.033 (2)	0.029 (2)	0.0030 (16)	0.0120 (17)	−0.0023 (16)
C23	0.036 (2)	0.033 (2)	0.036 (2)	−0.0006 (17)	0.0174 (19)	−0.0036 (17)
C24	0.043 (2)	0.033 (2)	0.054 (3)	−0.0021 (19)	0.022 (2)	0.001 (2)
C25	0.063 (3)	0.039 (2)	0.061 (3)	−0.007 (2)	0.023 (3)	0.008 (2)
C26	0.094 (4)	0.037 (3)	0.095 (4)	−0.015 (3)	0.039 (4)	0.002 (3)
C27	0.137 (6)	0.056 (3)	0.075 (4)	−0.043 (4)	0.020 (4)	−0.006 (3)
C28	0.094 (5)	0.061 (4)	0.077 (4)	−0.038 (3)	−0.001 (3)	−0.002 (3)
C29	0.047 (3)	0.038 (2)	0.049 (3)	−0.009 (2)	0.014 (2)	−0.005 (2)
C30	0.037 (2)	0.030 (2)	0.038 (2)	−0.0032 (17)	0.0140 (19)	−0.0029 (17)
C31	0.033 (2)	0.034 (2)	0.030 (2)	−0.0045 (17)	0.0102 (18)	−0.0050 (17)
C32	0.039 (2)	0.045 (2)	0.031 (2)	−0.0077 (19)	0.0024 (19)	−0.0081 (19)
C33	0.054 (3)	0.054 (3)	0.032 (2)	−0.013 (2)	0.008 (2)	0.006 (2)
C34	0.052 (3)	0.044 (3)	0.036 (2)	−0.009 (2)	0.009 (2)	0.0073 (19)
C35	0.033 (2)	0.034 (2)	0.029 (2)	0.0012 (16)	0.0122 (17)	−0.0037 (17)
C36	0.028 (2)	0.0298 (19)	0.032 (2)	−0.0002 (16)	0.0145 (17)	−0.0029 (16)
N1	0.047 (2)	0.0304 (18)	0.041 (2)	−0.0023 (15)	0.0219 (17)	−0.0038 (15)
N2	0.0369 (19)	0.0365 (18)	0.0322 (18)	−0.0009 (15)	0.0141 (15)	0.0008 (15)
N3	0.044 (2)	0.046 (2)	0.0354 (19)	0.0031 (16)	0.0165 (16)	0.0031 (16)
N4	0.040 (2)	0.0393 (18)	0.038 (2)	−0.0070 (15)	0.0195 (16)	−0.0039 (15)
N5	0.0335 (18)	0.0403 (19)	0.0281 (18)	−0.0082 (14)	0.0111 (15)	−0.0077 (14)
N6	0.0413 (19)	0.0359 (18)	0.0301 (18)	−0.0055 (15)	0.0110 (15)	0.0005 (15)
N7	0.043 (2)	0.0303 (18)	0.044 (2)	−0.0038 (15)	0.0193 (17)	−0.0005 (15)
N8	0.049 (2)	0.0352 (19)	0.042 (2)	−0.0151 (16)	0.0144 (17)	−0.0048 (16)
O1W	0.085 (6)	0.196 (10)	0.033 (4)	0.071 (7)	0.028 (4)	0.037 (5)
Cd	0.03979 (18)	0.03392 (17)	0.03215 (17)	−0.00684 (13)	0.01514 (13)	−0.00596 (13)
Cl1	0.0488 (7)	0.0659 (8)	0.0593 (7)	0.0030 (6)	0.0192 (6)	−0.0220 (6)
Cl2	0.0531 (7)	0.0642 (8)	0.0449 (7)	−0.0240 (6)	0.0109 (5)	−0.0129 (6)

### Geometric parameters (Å, °)

**Table:**

C1—N1	1.320 (5)	C20—H20	0.9300
C1—C2	1.393 (6)	C21—C22	1.395 (5)
C1—H1	0.9300	C21—H21	0.9300
C2—C3	1.360 (6)	C22—C36	1.404 (5)
C2—H2	0.9300	C22—C23	1.458 (5)
C3—C4	1.406 (5)	C23—N7	1.350 (5)
C3—H3	0.9300	C23—C30	1.401 (5)
C4—C18	1.408 (5)	C24—N7	1.321 (5)
C4—C5	1.449 (5)	C24—C29	1.426 (6)
C5—N3	1.353 (5)	C24—C25	1.499 (5)
C5—C12	1.395 (5)	C25—C26	1.480 (6)
C6—N3	1.331 (5)	C25—H25A	0.9700
C6—C11	1.415 (6)	C25—H25B	0.9700
C6—C7	1.492 (6)	C26—C27	1.488 (7)
C7—C8	1.523 (6)	C26—H26A	0.9700
C7—H7A	0.9700	C26—H26B	0.9700
C7—H7B	0.9700	C27—C28	1.407 (7)
C8—C9	1.493 (6)	C27—H27A	0.9700
C8—H8A	0.9700	C27—H27B	0.9700
C8—H8B	0.9700	C28—C29	1.501 (6)
C9—C10	1.509 (6)	C28—H28A	0.9700
C9—H9A	0.9700	C28—H28B	0.9700
C9—H9B	0.9700	C29—N8	1.314 (5)
C10—C11	1.497 (5)	C30—N8	1.352 (5)
C10—H10A	0.9700	C30—C31	1.452 (5)
C10—H10B	0.9700	C31—C32	1.403 (5)
C11—N4	1.322 (5)	C31—C35	1.409 (5)
C12—N4	1.357 (5)	C32—C33	1.354 (6)
C12—C13	1.451 (5)	C32—H32	0.9300
C13—C17	1.397 (5)	C33—C34	1.378 (6)
C13—C14	1.403 (5)	C33—H33	0.9300
C14—C15	1.367 (5)	C34—N6	1.321 (5)
C14—H14	0.9300	C34—H34	0.9300
C15—C16	1.396 (5)	C35—N6	1.361 (4)
C15—H15	0.9300	C35—C36	1.459 (5)
C16—N2	1.315 (5)	C36—N5	1.365 (4)
C16—H16	0.9300	Cd—N1	2.390 (3)
C17—N2	1.356 (4)	Cd—N2	2.445 (3)
C17—C18	1.453 (5)	Cd—N5	2.367 (3)
C18—N1	1.353 (5)	Cd—N6	2.532 (3)
C19—N5	1.330 (5)	Cd—Cl1	2.4886 (12)
C19—C20	1.386 (5)	Cd—Cl2	2.5067 (11)
C19—H19	0.9300	O1W—H1WA	0.87 (2)
C20—C21	1.368 (5)	O1W—H1WB	0.81 (2)
			
N1—C1—C2	123.3 (4)	N7—C24—C25	119.1 (4)
N1—C1—H1	118.4	C29—C24—C25	119.7 (4)
C2—C1—H1	118.4	C26—C25—C24	113.7 (4)
C3—C2—C1	119.3 (4)	C26—C25—H25A	108.8
C3—C2—H2	120.4	C24—C25—H25A	108.8
C1—C2—H2	120.4	C26—C25—H25B	108.8
C2—C3—C4	119.0 (4)	C24—C25—H25B	108.8
C2—C3—H3	120.5	H25A—C25—H25B	107.7
C4—C3—H3	120.5	C25—C26—C27	112.9 (4)
C3—C4—C18	118.1 (4)	C25—C26—H26A	109.0
C3—C4—C5	122.5 (4)	C27—C26—H26A	109.0
C18—C4—C5	119.4 (3)	C25—C26—H26B	109.0
N3—C5—C12	120.8 (4)	C27—C26—H26B	109.0
N3—C5—C4	118.7 (3)	H26A—C26—H26B	107.8
C12—C5—C4	120.4 (3)	C28—C27—C26	119.9 (5)
N3—C6—C11	120.9 (4)	C28—C27—H27A	107.3
N3—C6—C7	117.1 (4)	C26—C27—H27A	107.3
C11—C6—C7	121.9 (4)	C28—C27—H27B	107.3
C6—C7—C8	114.3 (4)	C26—C27—H27B	107.3
C6—C7—H7A	108.7	H27A—C27—H27B	106.9
C8—C7—H7A	108.7	C27—C28—C29	117.0 (5)
C6—C7—H7B	108.7	C27—C28—H28A	108.0
C8—C7—H7B	108.7	C29—C28—H28A	108.0
H7A—C7—H7B	107.6	C27—C28—H28B	108.0
C9—C8—C7	111.3 (4)	C29—C28—H28B	108.0
C9—C8—H8A	109.4	H28A—C28—H28B	107.3
C7—C8—H8A	109.4	N8—C29—C24	122.2 (4)
C9—C8—H8B	109.4	N8—C29—C28	117.4 (4)
C7—C8—H8B	109.4	C24—C29—C28	120.4 (4)
H8A—C8—H8B	108.0	N8—C30—C23	121.5 (3)
C8—C9—C10	110.8 (4)	N8—C30—C31	118.1 (3)
C8—C9—H9A	109.5	C23—C30—C31	120.4 (3)
C10—C9—H9A	109.5	C32—C31—C35	117.5 (3)
C8—C9—H9B	109.5	C32—C31—C30	122.8 (3)
C10—C9—H9B	109.5	C35—C31—C30	119.7 (3)
H9A—C9—H9B	108.1	C33—C32—C31	119.6 (4)
C11—C10—C9	112.8 (4)	C33—C32—H32	120.2
C11—C10—H10A	109.0	C31—C32—H32	120.2
C9—C10—H10A	109.0	C32—C33—C34	119.0 (4)
C11—C10—H10B	109.0	C32—C33—H33	120.5
C9—C10—H10B	109.0	C34—C33—H33	120.5
H10A—C10—H10B	107.8	N6—C34—C33	124.4 (4)
N4—C11—C6	121.5 (4)	N6—C34—H34	117.8
N4—C11—C10	118.2 (4)	C33—C34—H34	117.8
C6—C11—C10	120.3 (4)	N6—C35—C31	122.2 (3)
N4—C12—C5	121.2 (3)	N6—C35—C36	117.9 (3)
N4—C12—C13	118.6 (3)	C31—C35—C36	119.9 (3)
C5—C12—C13	120.2 (3)	N5—C36—C22	122.0 (3)
C17—C13—C14	117.7 (3)	N5—C36—C35	117.7 (3)
C17—C13—C12	119.5 (3)	C22—C36—C35	120.3 (3)
C14—C13—C12	122.7 (3)	C1—N1—C18	118.4 (4)
C15—C14—C13	119.7 (4)	C1—N1—Cd	123.5 (3)
C15—C14—H14	120.1	C18—N1—Cd	116.7 (2)
C13—C14—H14	120.1	C16—N2—C17	118.4 (3)
C14—C15—C16	118.4 (4)	C16—N2—Cd	125.1 (3)
C14—C15—H15	120.8	C17—N2—Cd	115.0 (2)
C16—C15—H15	120.8	C6—N3—C5	117.9 (3)
N2—C16—C15	123.5 (4)	C11—N4—C12	117.6 (3)
N2—C16—H16	118.3	C19—N5—C36	117.7 (3)
C15—C16—H16	118.3	C19—N5—Cd	121.3 (2)
N2—C17—C13	122.2 (3)	C36—N5—Cd	121.0 (2)
N2—C17—C18	117.5 (3)	C34—N6—C35	117.2 (3)
C13—C17—C18	120.3 (3)	C34—N6—Cd	127.4 (3)
N1—C18—C4	121.8 (3)	C35—N6—Cd	115.3 (2)
N1—C18—C17	118.4 (3)	C24—N7—C23	117.0 (3)
C4—C18—C17	119.8 (3)	C29—N8—C30	116.7 (3)
N5—C19—C20	123.7 (4)	H1WA—O1W—H1WB	108 (3)
N5—C19—H19	118.2	N5—Cd—N1	146.20 (10)
C20—C19—H19	118.2	N5—Cd—N2	86.77 (10)
C21—C20—C19	118.9 (4)	N1—Cd—N2	68.51 (11)
C21—C20—H20	120.6	N5—Cd—Cl1	101.33 (8)
C19—C20—H20	120.6	N1—Cd—Cl1	96.70 (8)
C20—C21—C22	119.6 (4)	N2—Cd—Cl1	161.14 (8)
C20—C21—H21	120.2	N5—Cd—Cl2	95.08 (8)
C22—C21—H21	120.2	N1—Cd—Cl2	107.93 (8)
C21—C22—C36	118.2 (3)	N2—Cd—Cl2	91.85 (8)
C21—C22—C23	122.2 (3)	Cl1—Cd—Cl2	104.21 (4)
C36—C22—C23	119.6 (3)	N5—Cd—N6	67.68 (10)
N7—C23—C30	121.4 (3)	N1—Cd—N6	84.11 (10)
N7—C23—C22	118.4 (3)	N2—Cd—N6	77.31 (11)
C30—C23—C22	120.1 (3)	Cl1—Cd—N6	89.93 (8)
N7—C24—C29	121.2 (4)	Cl2—Cd—N6	159.84 (8)

### Hydrogen-bond geometry (Å, °)

**Table:**

*D*—H···*A*	*D*—H	H···*A*	*D*···*A*	*D*—H···*A*
O1W—H1WB···Cl2^i^	0.81 (2)	2.79 (7)	3.326 (7)	126 (7)

Symmetry codes: (i) −*x*+1, −*y*+1, −*z*.
